# The Utility of MRI in the Diagnosis of Takayasu Arteritis

**DOI:** 10.1155/2017/7976165

**Published:** 2017-10-08

**Authors:** Marian Gaballah, Rachelle Goldfisher, John B. Amodio

**Affiliations:** ^1^Department of Radiology, Hofstra Northwell School of Medicine, 500 Hofstra Boulevard, Hempstead, NY 11549, USA; ^2^Department of Radiology, Cohen's Children's Medical Center, 269-01 76th Avenue, New Hyde Park, NY 11040, USA

## Abstract

Takayasu Arteritis (TA) is an inflammatory disorder involving the thoracoabdominal aorta and its branches and the pulmonary arteries, with eventual vascular stenosis, occlusion, or aneurysm formation. Conventional angiography has been the reference imaging standard for diagnosis of TA. The purpose of this case report is to demonstrate the utility of MR imaging and MR angiography in the diagnosis of Takayasu Arteritis in a pediatric patient. The patient is a 15-year-old female patient presenting with anemia, hypertension, and acute kidney injury. Initial chest CT demonstrated ectasia of the ascending and focal stenosis of the descending thoracic aorta, prompting further evaluation with MRI and MRA. MRI/MRA demonstrated mural thickening with luminal stenosis of the aorta and aortic branch vessels. These imaging findings were suggestive of a large vessel arteritis and along with the clinical presentation and laboratory abnormalities the diagnosis of Takayasu Arteritis was suggested. Several case series in adults have described the cross-sectional findings of TA. However, this case report demonstrates the utility of MRI/MRA in the evaluation of TA in children and in the course of follow-up, as it provides a noninvasive method for evaluating a child without ionizing radiation or iodinated contrast.

## 1. Introduction

Takayasu arteritis (TA) is an inflammatory disorder involving the thoracoabdominal aorta and its branches, as well as the pulmonary arteries. This vasculitis results in granulomatous inflammation of the vessel wall with intimal proliferation and fibrosis of the media and adventitia. TA affects patients mostly in the second and third decades of life, with 80–90% of patients affected with TA being female [1].

Conventional angiography has been the reference standard for imaging TA and allows evaluation of the vessel lumen. However, angiography is invasive and provides ionizing radiation [2, 3], which are particularly important considerations in the evaluation of a child. Additionally, early inflammatory changes to the vessel include mural edema, wall thickening, and wall enhancement, which may be demonstrated on cross-sectional imaging, allowing earlier detection of disease prior to the development of stenosis, occlusion, or aneurysms [1, 4]. We present a case of Takayasu Arteritis in a pediatric patient, where MRI and MRA were utilized in the initial evaluation of extent and severity of the inflammatory process, as well as during interval follow-up of the disease.

## 2. Case History

**Figure 1:**
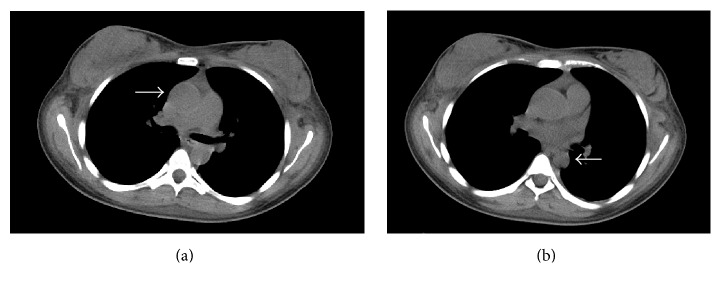
Noncontrast CT of the chest demonstrated (a) ectasia of the ascending aorta, measuring 3.4 cm, and (b) focal narrowing of the descending aorta, measuring 1.7 cm, raising concern for an aortitis in the setting of glomerulonephritis. The white arrow in (a) represents the ascending thoracic aorta and in (b) the descending thoracic aorta.

15-year-old female with a remote medical history of acute rheumatic fever as a child presents with a hemoglobin of 6.5. Labs on presentation were notable for acute kidney injury with a creatinine of 4.9. The patient was also hypertensive, with a systolic blood pressure of 140. Further work-up demonstrated a combination of microcytic and normocytic anemia. Renal sonogram was notable for mildly echogenic kidneys, with subsequent renal biopsy demonstrating rapidly progressive glomerulonephritis. Unenhanced CT of the chest was then obtained to evaluate intrathoracic disease in the presence of renal failure and concern for vasculitis (Figure 1).

Further evaluation was notable for a positive ANA titer with a homogeneous pattern, ESR level of 170, and CRP level of 56.4.

**Figure 2:**
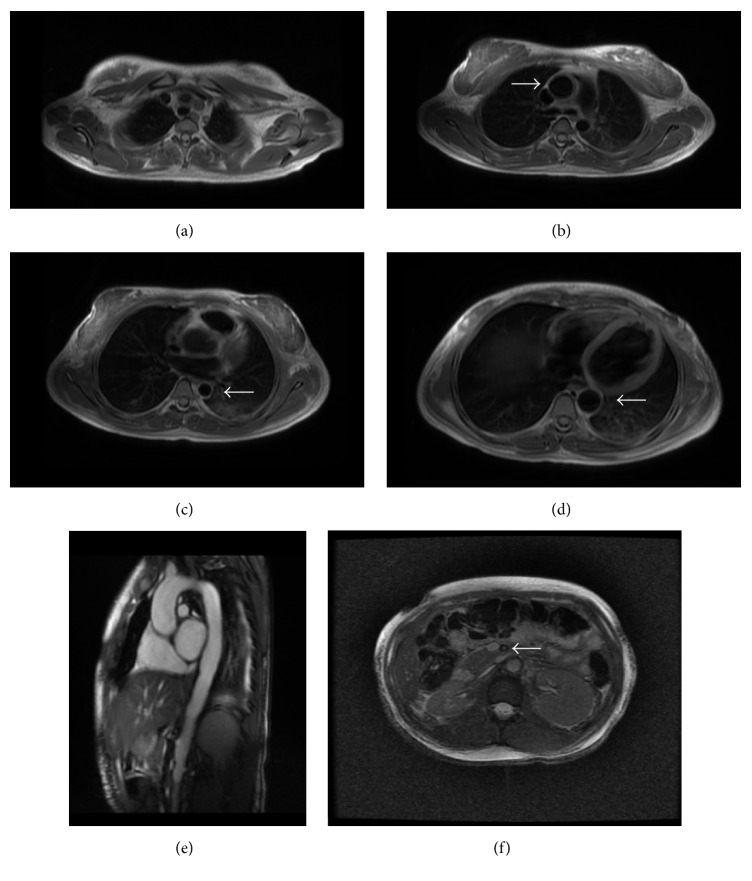
MRI/MRA through the chest, abdomen, and pelvis was obtained. (a) Axial double inversion recovery sequence through the upper chest demonstrates mural thickening of the brachiocephalic, left common carotid, and left subclavian arteries. (b) Axial double inversion recovery sequence demonstrates mural thickening of the ascending aorta which measures 3.4 cm in diameter. (c) Axial double inversion recovery sequence demonstrates a focal narrowing of the descending aorta, measuring 2.2 cm. (d) Inferior to the focal narrowing visualized in (c), the aorta measures 3.2 cm on axial double inversion recovery sequence. (e) Sagittal oblique cine-FIESTA sequence demonstrates the undulating contour of the abdominal aorta. (f) Axial T2 FIESTA fat saturated sequences through the abdomen demonstrate wall thickening and mural narrowing of the superior mesenteric artery. The white arrow represents in (b) ascending thoracic aorta; (c) and (d) descending thoracic aorta; (f) SMA.

MRI and MRA of the chest, abdomen, and pelvis were obtained to delineate the severity and extent of the aortitis and evaluate inflammatory involvement of additional vessels (Figure 2).

Clinical history, imaging findings, and laboratory abnormalities were attributed to Takayasu Arteritis. The patient was initially treated with several doses of pulse dose steroids, biologic therapy, and continued on steroid therapy and methotrexate. However, she had subsequent readmissions for steroid-induced pancreatitis and neutropenic fever.

**Figure 3:**
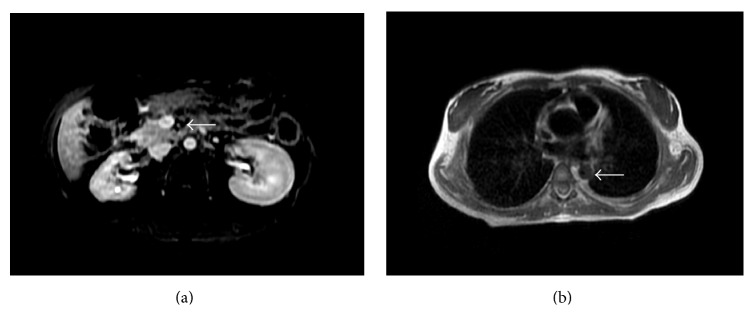
MRI and MRA of the abdomen and pelvis were obtained. (a) LAVA postcontrast sequence demonstrates mural thickening and marked luminal narrowing of the superior mesenteric artery. (b) Axial double inversion recovery sequence demonstrates increased focal narrowing of the descending thoracic aorta, measuring 1.6 cm in diameter. The white arrow represents in (a) SMA and in (b) descending thoracic aorta.

Approximately 3 months following initial diagnosis, the patient returned with intermittent bloody diarrhea. Repeat MRI/MRA was obtained at this time to evaluate for worsening vasculitis and sequelae of the inflammation (Figure 3).

## 3. Discussion

The acute phase of TA results in vessel wall thickening, mural edema, and mural enhancement. During the pulseless/occlusive phase, signs and symptoms result from vascular stenosis or occlusion and are dependent upon which vessels are involved. Stenosis is the most common complication of the aorta and most commonly involves the descending thoracic aorta and abdominal aorta, although aortic occlusion or dilation may also occur. Lesions of the branch vessels commonly involve the proximal portion of the vessel. Stenosis is the most common lesion of the aortic branch vessels and commonly involves the common carotid and subclavian arteries. The most frequently involved abdominal aortic branch vessel is the renal artery, although the most common anterior abdominal aortic branch vessel involved is the superior mesenteric artery. Collateral vessels may also develop in response to vessel stenosis or occlusion [5, 6].

Conventional angiography has been the reference standard for imaging in Takayasu Arteritis as it allows evaluation of the vessel lumen. However, inflammation in early vasculitis may manifest as vessel wall thickening, mural enhancement, or mural edema, rather than vascular stenosis, occlusion, or aneurysm [1–4]. This is best demonstrated by MRI/MRA. Angiography demonstrates vascular stenosis, although it cannot differentiate narrowing secondary to acute mural inflammation from that secondary to chronic transmural fibrosis [3]. Additionally, in the evaluation of children in particular, the invasive nature of angiography, the ionizing radiation administered, and the dose of iodinated contrast required must be considered [2, 3].

Although contrast enhanced CT will demonstrate mural thickening and hyperenhancement, intravenous contrast within the lumen may provide less contrast between the enhancing vessel wall and the contrast within the lumen. MR imaging provides the ability to suppress flowing blood on black-blood sequences, increasing contrast between lumen and wall, better demonstrating mural thickening and enhancement. MR imaging may also depict mural thrombi. T1-weighted sequences allow visualization of the thickened intima and media. The degree of aortic stenosis and/or dilation, as well as the extent of disease involvement, can be evaluated on T1-weighted spin-echo axial and sagittal oblique MR sequences [4]. Dilation of the ascending aorta can lead to aortic regurgitation which may be visualized using cine MR sequences [3, 4, 7]. Additionally, MR imaging allows for visualization of the vessel of interest in any desired plane. Disadvantages of MR imaging in evaluation of TA are difficulty visualizing small branch vessels and poor visualization of calcification [3].

Several studies have evaluated the sensitivity of MRA in detecting the vascular lesions of TA in comparison with conventional angiography. Kumar et al. described the use of 3D time of flight MR angiography and demonstrated an accuracy of 86%, sensitivity of 91%, and a specificity of 81% in detection of aortic arch and aortic arch branch vessel lesions, as well as accurately determining severity of the lesion in comparison with angiography. This study also described an accuracy of 92%, sensitivity of 97%, and specificity of 88% for identifying lesions of the abdominal aorta and the visceral branches, as well as accurately determining severity of the lesion compared with angiography. Given that patients with TA had more than one lesion, even in patients with false negative results, the diagnosis of TA was accurately made, allowing MRA to accurately diagnose TA in all patients in the study. False positives in this study were due to overestimation of lesion severity or diagnosis of a lesion in a normal vessel [8]. Yamada et al. demonstrated an accuracy, sensitivity, and specificity of 100% for detecting lesions of the thoracoabdominal aorta and major branch vessels using enhanced MR angiography, as well as for diagnosing TA. This study reported 98% accuracy for determining severity of detected lesions [9].

Choe et al. demonstrated that mural hyperenhancement, defined as enhancement hyperintense to myocardial enhancement, implied active arteritis. Additionally, this study proposed that aortic wall thickness alone may reflect the presence of disease, as patients with acute or chronic active TA demonstrated aortic wall thickness of 5–7 mm while those with inactive TA demonstrated wall thickness less than 4 mm. Disease activity determination using contrast enhanced MR imaging was concordant with clinical findings in 88.5% of patients, with ESR in 92.3% of patients, and with CRP in 84.6% of patients. There was a significant difference in aortic wall enhancement as well as aortic wall thickening between patients with a high ESR and those with a normal ESR [4].

In chronic TA, increased accumulation and delayed washout of contrast from the thickened aortic wall are suggestive of persistent inflammation, and MRI may be more sensitive than ESR or CRP levels in these cases [4].

The case presented demonstrated imaging findings suggestive of a chronic arteritis. The clinical presentation, however, was suggestive of an acute process. Given the lack of prior imaging studies, this case may represent an acute exacerbation of a chronic arteritis.

To conclude, the utility of MRI and MRA in detecting vessel wall thickening, enhancement, and mural edema and therefore allowing an early diagnosis of Takayasu Arteritis has been described in adults. Additionally, the lack of ionizing radiation as well as the noninvasive nature of this imaging study plays an integral role for children undergoing work-up for TA and in interval follow-up imaging.
